# Variation in the *RAD51 *gene and familial breast cancer

**DOI:** 10.1186/bcr1415

**Published:** 2006-06-08

**Authors:** Felicity Lose, Paul Lovelock, Georgia Chenevix-Trench, Graham J Mann, Gulietta M Pupo, Amanda B Spurdle

**Affiliations:** 1 Cancer and Cell Biology Division, Queensland Institute of Medical Research, Brisbane, Queensland, Australia; 2 School of Medicine, Central Clinical Division, University of Queensland, Royal Brisbane Hospital, Brisbane, Queensland, Australia; 3 School of Molecular and Microbial Sciences, University of Queensland, Brisbane, Queensland, Australia; 4 Westmead Institute for Cancer Research, University of Sydney at Westmead Millennium Institute, Westmead Hospital, Westmead, New South Wales, Australia

## Abstract

**Introduction:**

Human RAD51 is a homologue of the *Escherichia coli *RecA protein and is known to function in recombinational repair of double-stranded DNA breaks. Mutations in the lower eukaryotic homologues of RAD51 result in a deficiency in the repair of double-stranded DNA breaks. Loss of RAD51 function would therefore be expected to result in an elevated mutation rate, leading to accumulation of DNA damage and, hence, to increased cancer risk. RAD51 interacts directly or indirectly with a number of proteins implicated in breast cancer, such as BRCA1 and BRCA2. Similar to *BRCA1 *mice, *RAD51*^-/- ^mice are embryonic lethal. The *RAD51 *gene region has been shown to exhibit loss of heterozygosity in breast tumours, and deregulated RAD51 expression in breast cancer patients has also been reported. Few studies have investigated the role of coding region variation in the *RAD51 *gene in familial breast cancer, with only one coding region variant – exon 6 c.449G>A (p.R150Q) – reported to date.

**Methods:**

All nine coding exons of the *RAD51 *gene were analysed for variation in 46 well-characterised, *BRCA1/2*-negative breast cancer families using denaturing high-performance liquid chromatography. Genotyping of the exon 6 p.R150Q variant was performed in an additional 66 families. Additionally, lymphoblastoid cell lines from breast cancer patients were subjected to single nucleotide primer extension analysis to assess *RAD51 *expression.

**Results:**

No coding region variation was found, and all intronic variation detected was either found in unaffected controls or was unlikely to have functional consequences. Single nucleotide primer extension analysis did not reveal any allele-specific changes in *RAD51 *expression in all lymphoblastoid cell lines tested.

**Conclusion:**

Our study indicates that *RAD51 *is not a major familial breast cancer predisposition gene.

## Introduction

Hereditary breast cancer accounts for around 5–10% of all breast cancer cases, while the other 90–95% is assumed to be 'sporadic', with no apparent family history. A large proportion of familial breast cancer (<40%) can be attributed to mutations in the high-risk genes *BRCA1 *and *BRCA2 *[1]. Additional breast cancer genes have been discovered, largely through disease syndromes displaying a predisposition for breast cancer. Breast cancer in families with syndromes such as Li-Fraumeni syndrome (resulting from *p53 *gene mutations) [2] and Cowden syndrome (the mutated *PTEN *gene) [3], however, are each estimated to account for less than 1% of hereditary breast cancer, and mutations in *ATM *(the gene mutated in ataxia telangiectasia) and *CHEK2 *are also predicted to account for only a small proportion of familial breast cancer [4,5]. The genetic basis of the large majority of familial breast cancer therefore remains unaccounted for.

It is well known that deficiencies in DNA repair can lead to carcinogenesis. Double-stranded DNA breaks (DSBs) may be the most detrimental form of DNA damage because, if left unrepaired, the detection of broken chromosomes will lead to cell death. Additionally, if DSBs are repaired improperly, they can result in chromosomal translocations and cancer [6]. Central to the repair of DSBs by homologous recombination is RAD51, a homologue of the *Escherichia coli *DNA repair protein, RecA. RAD51 functions in DNA repair by mediating homologous pairing and strand exchange reactions [7], and its importance is further supported by the presence of many highly conserved orthologues [8].

RAD51 interacts (directly or indirectly) with a large number of proteins involved in DNA repair and the cell cycle, among others, as reviewed by Richardson [9]. Interestingly, four of these proteins – BRCA1, BRCA2, p53 and ATM – have been shown to be breast cancer predisposition genes in high-risk families. Additionally, *RAD51*^-/- ^mice are embryonic lethal, similar to *BRCA1*^-/- ^mice [10]. Alteration in either the expression or protein structure of RAD51 could therefore have similar deleterious effects on these essential pathways, leading to breast cancer.

Besides the interactions of RAD51 with key players in breast tumourigenesis, there is additional evidence to support a role for RAD51 in breast cancer. The *RAD51 *gene is located at chromosome position 15q15.1 [11], a region shown to exhibit loss of heterozygosity in a large range of cancers, including those of the lung, the colorectum and the breast [12]. Specifically, 70% of breast tumours (from subjects with an unknown family history) [12] and 32% of sporadic (nonfamilial) breast cancers have been found to exhibit loss of heterozygosity of this region [13]. RAD51 expression has also been found altered in both primary tumours and cancer cell lines. *RAD51 *mRNA expression in 16/16 of tumours from *BRCA1/2 *mutation-negative familial breast cancer patients was found to be one-half of that of the BT-474 breast cancer cell line [14], and protein levels were found to be decreased in 30% of breast tumours from a combination of sporadic and high-risk breast cancer patients [15].

In contrast, there are reports of increased RAD51 expression in tumours and cancer cell lines. Ma and colleagues [16] detected an increase in *RAD51 *mRNA expression in Ductal Carcinoma *In Situ*-Invasive Ductal Carcinoma transition and high-tumour-grade breast cancers, while it has also been found that overexpression of RAD51 correlates with histological grading of Invasive Ductal Carcinoma [17]. There are numerous reports of RAD51 overexpression in a large range of cancer cell lines, including cervical cancer, prostate cancer and breast cancer [18].

These RAD51 expression data are difficult to reconcile with a suppressor function, as suggested by the loss of heterozygosity data and knockout mouse data. Instead, the data suggest that any dysregulation of RAD51 may be associated with downstream biological and clinical effects. Indeed, it has been demonstrated that homologous pairing and strand transfer, essential events in the repair of DSBs, require stoichiometric amounts of RAD51 such that overexpression or underexpression of RAD51 results in a lack of formation of structures fundamental in the DSB repair process [19].

Despite the presented evidence for a role for deregulated expression of RAD51 in DSB repair defects and/or breast cancer, there have been few studies assessing the effects of *RAD51 *gene variation on breast cancer risk. These have largely focused on assessing the risk associated with a polymorphic variation in *RAD51*. Although there is some evidence that the rare -135G>C variant in *RAD51 *is involved in modifying the *BRCA1/2*-mutation-positive breast cancer phenotype, studies focusing on the association between the *RAD51 *-135G>C and -172G>T variants and breast cancer risk using case-control analysis have shown little support for a significant association with breast cancer [20].

With regard to *RAD51 *mutations and cancer predisposition, three studies have used various mutation detection methodologies to screen *RAD51 *in breast cancer patients. An analysis of 93 early-onset breast cancer cases, 9% of which had a strong family history, revealed no coding region variation [21]. A second study in 2000 assessed a Japanese population of 45 well-characterised high-risk breast cancer patients using single-strand conformation polymorphism (SSCP) analysis and detected one relatively rare variant, an arginine for glutamine substitution at amino acid 150 (c.449G>A) [22]. No other sequence alterations were detected in the Japanese study. Rapakko and colleagues [23] recently used conformation-sensitive gel electrophoresis to investigate 74 high-risk and 52 moderate-risk breast and/or ovarian cancer families of Finnish descent. All of the variation detected was noncoding and a majority was found in both cases and controls. It has been reported, however, that the sequence variation detection sensitivity of conformation-sensitive gel electrophoresis and SSCP is around 75% and 60–85%, respectively [24,25], and it is possible that some variation was not detected. We therefore sought to determine whether there is a role for variation in the entire *RAD51 *coding region (and surrounding intronic regions) in index cases from a well-characterised set of 49 *BRCA1/2 *mutation-negative breast cancer families, using the highly sensitive technique denaturing high-performance liquid chromatography (DHPLC). These families included a subset of families where cancer-affected individuals were shown to share a haplotype around the *RAD51 *locus. We also screened index cases from 71 families specifically for the previously reported *RAD51 *p.Arg150Gln variant [22].

## Methods

### Participants

Multiple-case breast cancer families were ascertained through the Kathleen Cuningham Foundation Consortium for Research into Familial Breast Cancer [26]. Ethics approvals were obtained from the Ethics Committees of the Peter MacCallum Cancer Institute and the Queensland Institute of Medical Research, and all participants gave written informed consent.

Inclusion criteria for this study included the following seven situations: criterion 1, the family contained four or more cases of breast cancer or contained at least three breast cancer cases, one of which possessed 'high-risk' features such as age at onset <40 years of age, the presence of male breast cancer in the family, bilateral breast cancer or ovarian and breast cancer in the same subject; criterion 2, at least two of the breast cancer cases in the family were alive, along with four or more living, unaffected, female first-degree or second-degree relatives over 18 years of age; criterion 3, three or more blood samples from breast cancer cases (on the same side of the family) were available; criterion 4, no pathogenic mutation in *BRCA1 *or *BRCA2 *had been identified in the family; criterion 5, no variants of unknown pathogenic significance in *BRCA1 *or *BRCA2 *had been identified in the family; criterion 6, a total of 11 or more blood samples from the family were available for genotyping analysis; and criterion 7, where data were available for a limited number of families, *RAD51 *haplotype sharing analysis (D15S1012, D15S994 and D15S978 located within 2 Mb of RAD51) showed evidence of a haplotype shared by affected participants in the *RAD51*-containing region, 15q15.1.

Fifty per cent of index cases (the youngest available breast cancer case) had been tested by diagnostic laboratories for mutations in *BRCA1 *and *BRCA2 *by full sequencing. There were no differences in allele frequency for any *RAD51 *variant when cases screened for *BRCA1/2 *mutation were compared with the total breast cancer sample.

A total of 73 index cases from 71 families fitting the first four criteria were genotyped for the *RAD51 *exon 6 c.449G>A (p.Arg150Gln) variant. A total of 51 index cases from 46 families fitting all seven criteria were screened for variation across the entire *RAD51 *coding region. A small overlap of five individuals from five families was analysed using both approaches. Multiple index cases were screened from a given family when it contained two cases with the same age at onset of breast cancer. For families screened across the entire *RAD51 *coding region, four families also contained individuals with either testicular or ovarian cancers and, if available, these subjects were also screened for variation in *RAD51 *due to the high expression of RAD51 in these tissues [22].

A control population of one of each pair of 93 unrelated, female, monozygotic twins (from a sample of 3,348 twin pairs described previously) was analysed to determine the control frequency of any variation detected in the *RAD51 *gene in this study. Individuals were almost exclusively of European origin and had been recruited through the Australian Twin Registry [27].

### *RAD51 *variation analysis

The *RAD51 *c.449G>A (p.Arg150Gln) variant was screened using PCR and restriction fragment length polymorphism analysis. Forward primer AAGATGTCATGAGGAGCTTGG and reverse primer GCCATAGTCTCTCTTATCTAAACCAG spanned the *Msp*I site present in the wildtype sequence (and lost in the A allele). PCR reactions were carried out in a 20 μl mixture containing 15 ng genomic DNA, 800 nM each primer, 200 μM each dATP, dCTP, dGTP and dTTP (Promega, Madison, WI, USA), 1.5 mM MgCl_2_, 1 × PCR buffer and 1 U Ampli*Taq *Gold polymerase (PE Applied Biosystems, Foster City, CA, USA). PCR amplification conditions were 5 minutes initial denaturation at 94°C, 30 cycles of 20 seconds at 94°C/20 s at 55°C/20 s at 72°C, and 10 minutes final extension at 72°C. *Msp*I (NEB, Ipswich, MA, USA) digestion (as specified by the manufacturer) of the 205 bp PCR product yielded fragments of 89 bp and 116 bp for the wildtype G allele, with a single fragment of 205 bp for the A allele. Digested PCR products were separated on a 4% Nusieve agarose gel.

Primers encompassing the 10 exons of *RAD51 *(and surrounding intronic regions) (Hs.446554; Unigene, NCBI) were designed using Primer3 software [28] (Table 1). PCR reactions were carried out in a 20 μl mixture containing, at final volume, 15 ng genomic DNA, 20–40 pmol each primer, 200 μM each dATP, dCTP, dGTP and dTTP (Promega), 1.5–2 mM MgCl_2_, 1 × PCR buffer and 1 U Ampli*Taq *Gold polymerase (PE Applied Biosystems). For some fragments, 0.5–1 M betaine was utilised to improve PCR and/or DHPLC profiles. The 'touchdown' PCR amplification conditions are detailed in Table 1. All products (and H_2_O controls) were first visualised on a 1.5% agarose gel before denaturation by heating to 95°C for 5 minutes and cooling to 60°C over a period of 30 minutes.

Samples were analysed on a Varian Helix DHPLC system (Varian, Palo Alto, CA, USA) at the recommended melt temperature(s) as determined by the Stanford DHPLC Melt Program [29] (detailed in Table 1). Analysis of the results was carried out using Star Workstation Reviewer software (version 5; Varian) and any aberrant or shifted profiles were reamplified for repeat by DHPLC before being sequenced using the Big-Dye version 3.1 sequencing chemistry and PE Applied Biosystems 377 sequencer.

### Allelic expression analysis (single nucleotide primer extension)

Lymphoblastoid cell lines (LCLs) were derived from individuals previously assessed for *RAD51 *variation and found to possess common single nucleotide polymorphisms (SNPs). All lines were grown in RPMI 1640 media with 10% foetal calf serum and 1% penicillin/streptomycin. RNA was extracted from LCLs using TriReagent (Sigma Chemicals, Perth, WA, Australia) according to the manufacturer's instructions. The RNA quality was checked by northern gel analysis and 1 μg each sample was placed in a reverse transcription-PCR reaction using Superscript III RNAse H^- ^Reverse Transcriptase, according to the manufacturer's instructions (Invitrogen, Carlsbad, CA, USA). PCR was then performed using the *RAD51 *exon 10c primers (Table 1), and the resulting product was purified using a Qiagen PCR purification kit (Qiagen, Hilden, Germany).

Primers located directly adjacent to the exon 10 c.1020*+718G>A SNP (forward primer, TTAAGTCCAGCTTGGCCAAG; and reverse primer, TGTAGAGATGGGATTTCACCA) were then used in single-cycle PCR reactions incorporating radiolabelled dNTPs. Both forward and reverse reactions were performed to verify results. Radiolabelled fragments were separated on a 10% denaturing acrylamide gel, and were visualised by autoradiography [30]. Densitometric analysis was performed using Fujifilm Multigauge 2003 software (Fuji Photo Film Co., Tokyo, Japan).

## Results

The c.449G>A (p.Arg150Gln) variant was not detected in any of the 71 families genotyped directly for this variant, nor was it detected by DHPLC screening across this region in an additional 41 families.

DHPLC analysis of 51 index breast cancer cases from 46 families with a strong family history revealed 13 different sequence variations, all of which were located in noncoding regions (Table 2).

The intron 7 and intron 8 variants have not been previously described. The intron 7 c.644+57G>T variant was not found in control subjects. This variant is not thought to be pathogenic, however, because, although the G nucleotide is conserved in the chimpanzee, the mouse and the rat [31], the variant is deeply intronic and is not predicted to alter splicing using the SpliceSiteFinder prediction program [32]. The intron 8 (c.775-41G>C) variant was not tested for in controls, but it is also unlikely to be functional due to its deep intronic location and the lack of predicted splicing effects.

All of the remaining five variants occurred at greater frequency in controls than cases. The intron 2 (c.97+110A>G) and intron 4 (c.344-36T>G) variants appeared to occur on the same haplotype in all cases and controls. Variants detected in the 5' untranslated region and the 3' untranslated region were common SNPs that have been reported previously, and were found in this population at the expected frequencies.

To assess the possibility that allelic imbalance of *RAD51 *expression may occur in breast cancer patients, we chose a common exonic SNP – exon 10 c.1020*+718G>A – as a marker of *RAD51 *gene expression, and we assessed four LCLs from individuals heterozygous for these variants using single nucleotide primer extension. The experiment was performed in both forward and reverse directions and was conducted twice, allowing verification of results. In all LCLs tested there was no evidence of one allele being favourably expressed over the other allele (data not shown).

## Discussion

RAD51 plays a central and critical role in the DNA repair process and also interacts with a number of proteins involved in important pathways, such as cell-cycle regulation and DNA damage signalling. Four studies have investigated the coding region of *RAD51 *for variation in breast cancer to date. Bell and colleagues [21] screened 93 early-onset (<40 years) breast cancer cases, 9% of which had a strong family history, for mutations in *RAD51*. A yeast-based protein truncation assay revealed no truncating mutations, and sequencing of a subset of 27 individuals with age at onset <30 years revealed no coding region variation. In the same study, a protein truncation assay of 15 breast cancer cell lines also revealed no truncating mutations.

Schmutte and colleagues [33] screened the *RAD51 *coding region and the surrounding intron/exon boundaries in 41 breast carcinomas (family history unknown) by SSCP but found no mutations.

There has been one report of a missense variant in *RAD51 *being detected in a breast cancer population to date. In a Japanese population of 20 breast cancer cases with a strong family history and 25 individuals with other high-risk factors such as bilateral breast cancer, and so on, a G>A nucleotide substitution was detected by SSCP in two individuals with bilateral breast cancer, which results in an arginine residue being substituted for a glutamine residue at amino acid position 150 of RAD51 [22]. This was an interesting finding as glutamine and arginine possess quite different physical properties that could be predicted to affect the RAD51 protein. No other coding region variation was detected, but the sensitivity of SSCP to detect variation is reported as only 60–85% [25].

In the present study we failed to detect the *RAD51 *c.449G>A (p.R150Q) variant in a large, well-characterised Australian familial breast cancer sample set. Our study assessed 112 high-risk breast cancer families for this variant, with our inclusion criteria (see Methods, first inclusion criterion) being similar to those of Kato and colleagues [22] aside from the fact that the *BRCA1/2 *mutation status was not reported in the Japanese study. The p.R150Q variant has not been reported in the National Center for Biotechnology Information (NCBI) SNP database [34] but it appears that it may be a relatively rare population-specific variant, and is probably not relevant to breast cancer in the Australian population.

Rappako and colleagues [23] recently used conformation-sensitive gel electrophoresis to screen 74 high-risk and 52 moderate-risk Finnish breast and/or ovarian cancer families for variation in the *RAD51 *gene. The inclusion criteria for a 'high-risk' family was similar to the current study, but we specifically investigated breast cancer patients and several families that shared a haplotype in the *RAD51 *region. In addition, DHPLC has been reported to be a more sensitive technique for detecting sequence variation [24]. Similar to the current study, no coding region variation was detected.

We did not detect any coding region variation in the *RAD51 *gene using the highly sensitive mutation detection technique DHPLC. A number of intronic variants were found. All variants, however, were either present at a similar frequency in controls and/or were located deep in the intron, and were therefore predicted not to have any functional effects. Additionally, single nucleotide primer extension analysis did not reveal any change in expression of *RAD51 *in LCLs from breast cancer patients, indicating that there is little, if any, allelic effect on *RAD51 *expression due to known or undetected genetic variation.

## Conclusion

The aim of the present study was to attempt to elucidate a role for *RAD51 *as a high-risk breast cancer predisposition gene using DHPLC and well-characterised non-*BRCA1/2 *familial breast cancer patients. We did not detect any variation in the coding region of *RAD51*, which may further imply the importance of maintaining the structural integrity of such a vital component of the DNA repair pathway. Although other noncoding variation in the *RAD51 *gene may be involved in *BRCA1/2*-mutation-negative breast cancers, the absence of coding or untranslated region mutations in this set of well-characterised familial cases implies that *RAD51 *is not a major familial breast cancer predisposition gene.

## Abbreviations

bp = base pair; DHPLC = denaturing high-performance liquid chromatography; DSB = double-stranded DNA break; LCL = lymphoblastoid cell line; PCR = polymerase chain reaction; SNP = single nucleotide polymorphism; SSCP = single strand conformation polymorphism.

## Competing interests

The authors declare that they have no competing interests.

## Authors' contributions

FL carried out the screening of the gene and drafted the manuscript. PL performed the single nucleotide primer extension analysis, and GJM and GMP performed the haplotype sharing analysis. The Kathleen Cuningham Foundation Consortium for Research into Familial Breast Cancer recruited and collected individuals for the study. GC-T participated in the design and coordination of the study. ABS conceived the study, and participated in its design and coordination, and helped to draft the manuscript. All authors read and approved the final manuscript.

## Figures and Tables

**Table 1:** *RAD51 *PCR conditions

Exon	PCR fragment	Forward primer	Reverse primer	Annealing temperature (°C)^a^	Size (bp)	DHPLC temperature (°C)
1	1	GCAAGCGAGTAGAGAAGTGGA	AACTGCCGCTGAGCACTG	54	218	65
2	2	ATGGCCTTGGCTTTTCCTAA	GGCCCTGCCAGACATATTTA	54	391	57
3	3	TGGAACCAACTTCCCATCTC	TTCCCACTAATGCCTCCCTA	54	341	58
4	4	CTCTTCCCATTGCACACCTT	CACCTGGCCTTCCTCTATCTC	54	371	52, 57
5	5	TCTGATGAGCTCCAAGAACA	TGACATGGAAGGATTTTGAAG	51	344	58
6	6	AAGGGAATGCCTCCTTCCTA	CCAAACTAACCCTGGCAATC	54	314	59
7	7	CAAATTGCTCATCTGCCTG	TGAGGCACCGTTTAACAAGA	54	397	59
8	8	TGGTAAGGAAGGGACCAGAA	TGTGGCCATAGACACTCCAA	57	390	60
9	9	TCGTTATTTTGTGGGGGAAA	ACAGGGGAGAGGCATATCAA	54	447	58
10	10a	TTGGTGCTTTGGTCTGTGTC	ATACCCCTCCTCCAAAACCA	54	409	59
10	10b	CAGGAGACAGGTCAGTAGTCACA	AGGTTTGGCACAAGACTCCA	51	374	57
10	10c	TGATCTTGTGTAAGGGTTTGGTT	GCAATCTCGACTCACTGCAA	51	375	60
10	10d	GATAGCCTGAGGTGGGAGAA	TCTGCAAGTGGGACTTTCCT	54	319	60

^a^'Touchdown' PCR amplification conditions were as follows: denaturation at 94°C for 10 minutes, followed by two cycles of 94°C for 30 seconds, 30 seconds at the fragment annealing temperature (*T*_A_) + 6°, and 72°C for 30 seconds. The conditions remained the same for the rest of the PCR except for the annealing temperature, which consisted of two cycles at (*T*_A _+ 4°C), then two cycles at (*T*_A _+ 2°C) and, finally, 35 cycles at the *T*_A_. A final extension step was conducted at 72°C for 7 minutes.

**Table 2:** Variation in the *RAD51 *gene

Gene region	PCR fragment	Nucleotide change	Amino acid change	Number (%) of heterozygous families^a^	Number (%) of heterozygous controls
Exon 1 (5' UTR)	1	c.-135G>C	-	3/28 (10.7%)	N/D
Exon 1 (5' UTR)	1	c.-172G>T	-	16/33 (48.5%)	N/D
Intron 2	2	c.97+110A>G	-	3/46 (6.5%)	16/93 (17.2%)
Intron 3	4	c.226-33T>G	-	11/46 (23.9%)	23/93 (24.7%)
Intron 3	4	c.226-70T>A	-	20/46 (43.5%)	43/93 (46.2%)
Intron 3	4	c.226-72delA	-	2/46 (4.3%)	8/93 (8.6%)
Intron 3	4	c.226-33T>G and c.226-70T>A	-	4/46 (8.7%)	8/93 (8.6%)
Intron 3	4	c.226-33T>G and c.226-72delA	-	1/46 (2.1%)	0/93 (0%)
Intron 3	4	c.226-70T>A and c.226-72delA	-	1/46 (2.1%)	6/93 (6.5%)
Intron 4	5	c.344-36T>G	-	3/46 (6.5%)	16/93 (17.2%)
Exon 6	6	c.449G>A	p.R150Q^b^	0/46 (0%), 0/66 (0%)	N/D
Intron 7	7	c.644+57G>T	-	1/46 (2.2%)	0/93 (0%)
Intron 8	9	c.775-41G>C	-	1/46 (2.2%)	N/D
Exon 10 (3' UTR)	10b	c.1020*+502T>G	-	19/46 (41.3%)	N/D
Exon 10 (3' UTR)	10c	c.1020*+718G>A	-	18/44 (40.9%)	N/D
Exon 10 (3' UTR)	10d	c.1020*+927T>C	-	17/42 (40.4%)	N/D

UTR, untranslated region; N/D, not determined ^a^Values expressed as the number of heterozygous families because all related individuals shared the same genotype. ^b^Reported in Kato and colleagues [22].
